# Mapping Chronic Disease Burden: Linking Social Determinants of Health to Chronic Disease Prevalence in Michigan

**DOI:** 10.51894/001c.164366

**Published:** 2026-07-31

**Authors:** Taylor Read, Hannah Taylor, Rachelle Pichot, Patrick Cranor, Matthew Hysell, Maddi Massa

**Affiliations:** 1 Corewell Health Lakeland Hospitals

## 36

### Introduction

Social determinant of health is a term for all nonmedical factors affecting health. Prior studies have shown that social advantages are directly related to better health outcomes. Our population is 3 different regions in Michigan at Corewell Health sites labeled South, West and East.

### Objectives

Our research study aims to find the most frequently experienced social determinants of health affecting Michigan patients, especially as it pertains to diabetes, hypertension, asthma, CHF, CKD, COPD and ischemic heart disease.

### Methods

Data was retrospectively extracted from January 2024 – July 2025 regarding social determinants of health intake form asked to all admitted patients. An online dashboard was created using data extracted from Epic about the 7 diseases of interest as well as the following social determinants of health: area deprivation index, transportation, social isolation, housing and financial strain. All analyses were conducted in R studio with population level descriptive statistics reported as percentages and Pearson chi-square tests of independence for associations with the criteria for statistical significance set at α = .05 (two-tailed).

### Results

828,182 admitted patients were included in this period. Geographically, East accounted for 36.97%, South 7.89%, and West 55.14% of the data. Our population was 55.95% female and 75.37% white. The south region had the highest prevalence of all diseases except for asthma; East had the highest prevalence of asthma. Area deprivation index is a composite measure that assesses relative disadvantage with a higher score meaning greater deprivation. The relationship between disease prevalence and area deprivation index was significant *p* < 0.001. Transportation risk was more prevalent in COPD, asthma, and CHF. Patients with COPD and asthma reported the most financial strain risk. Among those with high housing risk, there was a higher-than-expected prevalence of diabetes and asthma.

### Conclusions

This analysis was performed on the largest health system in Michigan with three distinct service areas. Patients with lung-related diagnoses including COPD and asthma were frequently the most affected by social determinants of health. Additional evaluation is needed to study if directly supporting the patient’s affected social determinants of health can help decrease prevalence or disease burden on that population.

